# Ethyl 7-amino-1-cyclo­propyl-6-fluoro-8-meth­oxy-4-oxo-1,4-dihydro­quinoline-3-carboxyl­ate monohydrate

**DOI:** 10.1107/S1600536808002547

**Published:** 2008-01-30

**Authors:** Jia Pan, Li Yang, Zhi-Hua Mao, Ling-Ling Weng

**Affiliations:** a Department of Medicinal Chemistry, West China School of Pharmacy, Sichuan University, Chengdu 610041, People’s Republic of China; b The Center for Testing and Analysis, Sichuan University, Chengdu 610064, People’s Republic of China

## Abstract

In the title compound, C_16_H_17_FN_2_O_4_·H_2_O, the dihedral angle between the heterocyclic ring and the benzene ring is 5.77 (9)°, that between the heterocycle and the ethoxy­carbonyl plane is 15.5 (1)°, and that between the heterocyclic ring and the cyclopropane ring is 67.75 (13)°. In the crystal structure, mol­ecules are linked into a ribbon-like structure along the *c* axis by N—H⋯O and O—H⋯O hydrogen bonds.

## Related literature

For general background, see: Fujita & Chiba (1998 ▶).
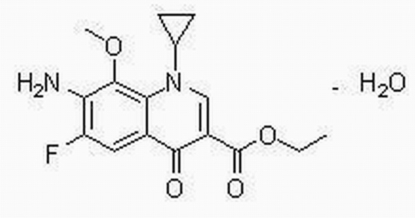


## Experimental

### 

#### Crystal data

C_16_H_17_FN_2_O_4_·H_2_O
*M* *_r_* = 338.33
Monoclinic, 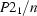

*a* = 10.096 (4) Å
*b* = 14.699 (5) Å
*c* = 11.028 (6) Å
β = 94.26 (4)°
*V* = 1632.0 (12) Å^3^
*Z* = 4
Mo *K*α radiation
μ = 0.11 mm^−1^
*T* = 291 (2) K
0.45 × 0.42 × 0.39 mm

#### Data collection

Enraf–Nonius CAD-4 diffractometer
Absorption correction: none
3157 measured reflections
3009 independent reflections
1741 reflections with *I* > 2σ(*I*)
*R*_int_ = 0.007
3 standard reflections every 300 reflections intensity decay: 0.8%

#### Refinement

*R*[*F* ^2^ > 2σ(*F* ^2^)] = 0.043
*wR*(*F* ^2^) = 0.125
*S* = 1.04
3009 reflections
235 parameters
H atoms treated by a mixture of independent and constrained refinement
Δρ_max_ = 0.20 e Å^−3^
Δρ_min_ = −0.19 e Å^−3^

### 

Data collection: *DIFRAC* (Gabe & White, 1993 ▶); cell refinement: *DIFRAC*; data reduction: *NRCVAX* (Gabe *et al.*, 1989 ▶); program(s) used to solve structure: *SHELXS97* (Sheldrick, 2008 ▶); program(s) used to refine structure: *SHELXL97* (Sheldrick, 2008 ▶); molecular graphics: *ORTEP-3 for Windows* (Farrugia, 1997 ▶); software used to prepare material for publication: *SHELXL97*.

## Supplementary Material

Crystal structure: contains datablocks global, I. DOI: 10.1107/S1600536808002547/ci2556sup1.cif

Structure factors: contains datablocks I. DOI: 10.1107/S1600536808002547/ci2556Isup2.hkl

Additional supplementary materials:  crystallographic information; 3D view; checkCIF report

## Figures and Tables

**Table 1:** Hydrogen-bond geometry (Å, °)

*D*—H⋯*A*	*D*—H	H⋯*A*	*D*⋯*A*	*D*—H⋯*A*
N1—H1*N*1⋯O1*W*^i^	0.91 (3)	2.15 (3)	2.930 (3)	143 (2)
N1—H2*N*1⋯O4^ii^	0.85 (3)	2.33 (3)	3.061 (3)	144 (2)
O1*W*—H1*W*⋯O2^iii^	0.84 (3)	2.13 (3)	2.916 (3)	155 (3)
O1*W*—H2*W*⋯O2	0.91 (3)	1.96 (3)	2.864 (3)	171 (3)

Symmetry codes: (i) 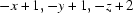; (ii) 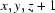; (iii) 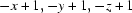.

**Fig. 1.**
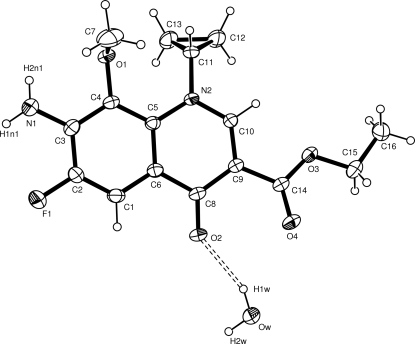
The molecular structure of the title compound, showing the atomic numbering scheme. Displacement ellipsoids drawn at the 30% probability level. The dashed line indicates a hydrogen bond.

**Fig. 2.**
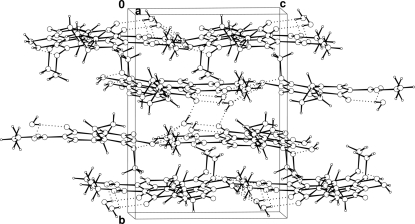
Crystal packing of the title compound, viwed down the a axis. Hydrogen bonds are shown as dashed lines.

## supplementary crystallographic information

### Comment

Quinolone antibacterials were found several decades ago, and some excellent antibacterials have been developed and used widely now (Fujita & Chiba, 1998). An interest in search of more potent antibacterial agents led us to design and synthesis a new type of quinoline derivatives. The title compound is one of the key intermediates and we report here its crystal structure.

The pyridinone ring is planar to within ±0.057 (2) Å (Fig. 1). The dihedral angle between the pyridine and benzene rings is 5.77 (9)°, and that between the pyridine and carboxylate plane is 15.5 (1)°. In the crystal structure, the molecules are linked into a ribbon like structure along the *c* axis (Fig. 2) by N—H···O and O—H···O hydrogen bonds (Table 1).

### Experimental

Ethyl 7-azido-1-cyclopropyl-6-fluoro-8-methoxyl-4-oxo-1,4-dihydroquinoline-3- carboxylate (2 g, 5.8 mmol), 5% Pd/C (0.4 g) were suspended in methanol (20 ml) and the mixture was hydrogenated at 303 K for 6 h. The reaction mixture was then filtered and concentrated under vacuum. The residue obtained was purified by silica gel chromatography. Single crystals suitable for X-ray analysis were obtained by slow evaporation of a acetyl acetate-chloroform (1.2:1 *v*/*v*) solution at room temperature.

### Refinement

The water H atoms were located in a difference Fourier map and refined isotropically. The remaining H atoms were placed in the calculated positions [C—H = 0.93 (aromatic) and 0.96 Å (methyl)] and refined in the riding-model approximation, with *U*_iso_(H) = 1.2*U*_eq_(aromatic-C) and 1.5*U*_eq_(methyl-C).

### Crystal data

**Table:**

C_16_H_17_FN_2_O_4_·H_2_O	*F*_000_ = 712
*M**_r_* = 338.33	*D*_x_ = 1.377 Mg m^−^^3^
Monoclinic, *P*2_1_/*n*	Mo *K*α radiation λ = 0.71073 Å
Hall symbol: -P 2yn	Cell parameters from 24 reflections
*a* = 10.096 (4) Å	θ = 4.5–7.4º
*b* = 14.699 (5) Å	µ = 0.11 mm^−^^1^
*c* = 11.028 (6) Å	*T* = 291 (2) K
β = 94.26 (4)º	Block, yellow
*V* = 1632.0 (12) Å^3^	0.45 × 0.42 × 0.39 mm
*Z* = 4	

### Data collection

**Table:**

Enraf–Nonius CAD-4 diffractometer	*R*_int_ = 0.007
Radiation source: fine-focus sealed tube	θ_max_ = 25.4º
Monochromator: graphite	θ_min_ = 2.3º
*T* = 291(2) K	*h* = −12→12
ω/2θ scans	*k* = 0→17
Absorption correction: none	*l* = −4→13
3157 measured reflections	3 standard reflections
3009 independent reflections	every 300 reflections
1741 reflections with *I* > 2σ(*I*)	intensity decay: 0.8%

### Refinement

**Table:**

Refinement on *F*^2^	Secondary atom site location: difference Fourier map
Least-squares matrix: full	Hydrogen site location: mixed
*R*[*F*^2^ > 2σ(*F*^2^)] = 0.043	H atoms treated by a mixture of independent and constrained refinement
*wR*(*F*^2^) = 0.125	*w* = 1/[σ^2^(*F*_o_^2^) + (0.0627*P*)^2^ + 0.1314*P*] where *P* = (*F*_o_^2^ + 2*F*_c_^2^)/3
*S* = 1.04	(Δ/σ)_max_ = 0.001
3009 reflections	Δρ_max_ = 0.20 e Å^−^^3^
235 parameters	Δρ_min_ = −0.19 e Å^−^^3^
Primary atom site location: structure-invariant direct methods	Extinction correction: none

### Special details

**Table:**

Geometry. All e.s.d.'s (except the e.s.d. in the dihedral angle between two l.s. planes) are estimated using the full covariance matrix. The cell e.s.d.'s are taken into account individually in the estimation of e.s.d.'s in distances, angles and torsion angles; correlations between e.s.d.'s in cell parameters are only used when they are defined by crystal symmetry. An approximate (isotropic) treatment of cell e.s.d.'s is used for estimating e.s.d.'s involving l.s. planes.
Refinement. Refinement of *F*^2^ against ALL reflections. The weighted *R*-factor *wR* and goodness of fit *S* are based on *F*^2^, conventional *R*-factors *R* are based on *F*, with *F* set to zero for negative *F*^2^. The threshold expression of *F*^2^ > σ(*F*^2^) is used only for calculating *R*-factors(gt) *etc*. and is not relevant to the choice of reflections for refinement. *R*-factors based on *F*^2^ are statistically about twice as large as those based on *F*, and *R*- factors based on ALL data will be even larger.

### Fractional atomic coordinates and isotropic or equivalent isotropic displacement parameters (Å^2^)

**Table:**

	*x*	*y*	*z*	*U*_iso_*/*U*_eq_	
F1	0.51891 (13)	0.59935 (11)	1.01305 (12)	0.0667 (4)	
O1	0.07128 (14)	0.68291 (10)	1.03507 (12)	0.0451 (4)	
O2	0.33328 (14)	0.56826 (12)	0.58213 (13)	0.0519 (4)	
O3	−0.04405 (16)	0.62649 (11)	0.41364 (13)	0.0552 (5)	
O4	0.16963 (18)	0.62837 (18)	0.37733 (15)	0.1007 (9)	
N1	0.3174 (3)	0.65375 (16)	1.14712 (18)	0.0514 (5)	
H1N1	0.385 (3)	0.6210 (17)	1.184 (2)	0.068 (9)*	
H2N1	0.248 (3)	0.6504 (18)	1.186 (2)	0.071 (9)*	
N2	0.01966 (16)	0.64366 (12)	0.77689 (13)	0.0374 (4)	
C1	0.3794 (2)	0.59542 (15)	0.83243 (19)	0.0436 (5)	
H1	0.4507	0.5774	0.7894	0.052*	
C2	0.3970 (2)	0.61005 (15)	0.95442 (19)	0.0433 (5)	
C3	0.2938 (2)	0.63708 (14)	1.02489 (17)	0.0397 (5)	
C4	0.1693 (2)	0.65127 (13)	0.96495 (17)	0.0352 (5)	
C5	0.1457 (2)	0.63418 (13)	0.83915 (17)	0.0333 (5)	
C6	0.2533 (2)	0.60753 (13)	0.77155 (17)	0.0358 (5)	
C7	0.0710 (3)	0.78037 (18)	1.0445 (3)	0.0786 (9)	
H7A	0.1564	0.8008	1.0778	0.118*	
H7B	0.0041	0.7990	1.0968	0.118*	
H7C	0.0523	0.8064	0.9653	0.118*	
C8	0.2389 (2)	0.59719 (14)	0.63888 (17)	0.0378 (5)	
C9	0.1107 (2)	0.62227 (14)	0.58234 (17)	0.0401 (5)	
C10	0.0092 (2)	0.64129 (14)	0.65419 (17)	0.0397 (5)	
H10	−0.0738	0.6536	0.6152	0.048*	
C11	−0.1031 (2)	0.65083 (17)	0.83913 (18)	0.0449 (6)	
H11	−0.1233	0.7109	0.8716	0.054*	
C12	−0.2192 (2)	0.5947 (2)	0.7935 (2)	0.0723 (9)	
H12A	−0.2086	0.5554	0.7242	0.087*	
H12B	−0.3070	0.6208	0.7971	0.087*	
C13	−0.1436 (2)	0.5712 (2)	0.9108 (2)	0.0627 (7)	
H13A	−0.1857	0.5830	0.9854	0.075*	
H13B	−0.0873	0.5177	0.9126	0.075*	
C14	0.0853 (2)	0.62535 (17)	0.44863 (19)	0.0508 (6)	
C15	−0.0802 (3)	0.6314 (2)	0.28436 (19)	0.0693 (8)	
H15A	−0.0528	0.5763	0.2449	0.083*	
H15B	−0.0360	0.6826	0.2493	0.083*	
C16	−0.2257 (2)	0.64228 (17)	0.2653 (2)	0.0589 (7)	
H16A	−0.2687	0.5904	0.2979	0.088*	
H16B	−0.2506	0.6471	0.1798	0.088*	
H16C	−0.2523	0.6964	0.3058	0.088*	
O1W	0.5509 (2)	0.44950 (16)	0.65115 (16)	0.0715 (6)	
H1W	0.606 (3)	0.446 (2)	0.598 (3)	0.091 (11)*	
H2W	0.486 (3)	0.488 (2)	0.621 (3)	0.097 (11)*	

### Atomic displacement parameters (Å^2^)

**Table:**

	*U*^11^	*U*^22^	*U*^33^	*U*^12^	*U*^13^	*U*^23^
F1	0.0396 (8)	0.1032 (12)	0.0559 (8)	0.0081 (7)	−0.0058 (6)	−0.0147 (8)
O1	0.0457 (9)	0.0567 (10)	0.0346 (7)	0.0092 (8)	0.0143 (6)	−0.0021 (7)
O2	0.0422 (9)	0.0766 (11)	0.0388 (8)	0.0138 (8)	0.0163 (7)	−0.0026 (7)
O3	0.0469 (10)	0.0891 (13)	0.0303 (8)	0.0030 (9)	0.0083 (7)	0.0013 (7)
O4	0.0523 (12)	0.215 (3)	0.0373 (10)	0.0254 (13)	0.0193 (9)	0.0150 (12)
N1	0.0482 (13)	0.0683 (15)	0.0378 (11)	0.0021 (11)	0.0036 (10)	−0.0036 (10)
N2	0.0332 (10)	0.0504 (11)	0.0298 (9)	0.0072 (8)	0.0109 (7)	0.0037 (7)
C1	0.0340 (12)	0.0527 (14)	0.0451 (13)	0.0016 (10)	0.0109 (10)	−0.0051 (10)
C2	0.0337 (12)	0.0528 (14)	0.0428 (12)	0.0019 (10)	−0.0008 (10)	−0.0034 (10)
C3	0.0449 (13)	0.0402 (12)	0.0344 (11)	−0.0043 (10)	0.0063 (9)	0.0002 (9)
C4	0.0364 (12)	0.0368 (11)	0.0340 (10)	0.0013 (9)	0.0121 (9)	0.0001 (9)
C5	0.0331 (11)	0.0336 (11)	0.0341 (10)	0.0011 (9)	0.0075 (8)	0.0031 (8)
C6	0.0337 (12)	0.0373 (12)	0.0373 (11)	−0.0026 (9)	0.0085 (9)	−0.0001 (8)
C7	0.097 (2)	0.0622 (18)	0.0805 (19)	0.0211 (17)	0.0311 (17)	−0.0148 (15)
C8	0.0384 (12)	0.0414 (12)	0.0355 (11)	−0.0007 (10)	0.0145 (9)	−0.0008 (9)
C9	0.0378 (12)	0.0518 (14)	0.0315 (11)	0.0038 (10)	0.0093 (9)	0.0018 (9)
C10	0.0350 (12)	0.0519 (14)	0.0327 (10)	0.0079 (10)	0.0069 (9)	0.0078 (9)
C11	0.0320 (12)	0.0659 (15)	0.0385 (11)	0.0082 (11)	0.0129 (9)	0.0041 (10)
C12	0.0403 (15)	0.130 (3)	0.0480 (14)	−0.0086 (15)	0.0124 (12)	0.0057 (15)
C13	0.0492 (15)	0.094 (2)	0.0469 (13)	−0.0127 (14)	0.0168 (11)	0.0119 (13)
C14	0.0444 (14)	0.0763 (17)	0.0331 (11)	0.0094 (12)	0.0123 (10)	0.0023 (11)
C15	0.0627 (18)	0.114 (2)	0.0311 (12)	0.0122 (16)	0.0048 (12)	−0.0030 (13)
C16	0.0602 (17)	0.0649 (17)	0.0508 (14)	0.0107 (13)	−0.0012 (12)	−0.0041 (12)
O1W	0.0600 (13)	0.1157 (18)	0.0396 (9)	0.0301 (12)	0.0077 (9)	−0.0035 (10)

### Geometric parameters (Å, °)

**Table:**

F1—C2	1.356 (2)	C7—H7B	0.96
O1—C4	1.381 (2)	C7—H7C	0.96
O1—C7	1.436 (3)	C8—C9	1.442 (3)
O2—C8	1.252 (2)	C9—C10	1.371 (3)
O3—C14	1.334 (3)	C9—C14	1.479 (3)
O3—C15	1.447 (3)	C10—H10	0.93
O4—C14	1.202 (3)	C11—C13	1.486 (3)
N1—C3	1.373 (3)	C11—C12	1.490 (3)
N1—H1N1	0.91 (3)	C11—H11	0.98
N1—H2N1	0.85 (3)	C12—C13	1.492 (4)
N2—C10	1.350 (2)	C12—H12A	0.97
N2—C5	1.408 (3)	C12—H12B	0.97
N2—C11	1.465 (3)	C13—H13A	0.97
C1—C2	1.361 (3)	C13—H13B	0.97
C1—C6	1.405 (3)	C15—C16	1.477 (3)
C1—H1	0.9300	C15—H15A	0.97
C2—C3	1.403 (3)	C15—H15B	0.97
C3—C4	1.391 (3)	C16—H16A	0.96
C4—C5	1.413 (3)	C16—H16B	0.96
C5—C6	1.418 (3)	C16—H16C	0.96
C6—C8	1.467 (3)	O1W—H1W	0.84 (3)
C7—H7A	0.96	O1W—H2W	0.91 (3)
			
C4—O1—C7	112.45 (17)	N2—C10—C9	125.34 (19)
C14—O3—C15	117.11 (18)	N2—C10—H10	117.3
C3—N1—H1N1	114.2 (16)	C9—C10—H10	117.3
C3—N1—H2N1	113.3 (18)	N2—C11—C13	117.9 (2)
H1N1—N1—H2N1	112 (2)	N2—C11—C12	118.3 (2)
C10—N2—C5	119.16 (17)	C13—C11—C12	60.20 (17)
C10—N2—C11	117.75 (17)	N2—C11—H11	116.3
C5—N2—C11	123.00 (15)	C13—C11—H11	116.3
C2—C1—C6	119.99 (19)	C12—C11—H11	116.3
C2—C1—H1	120.0	C11—C12—C13	59.79 (16)
C6—C1—H1	120.0	C11—C12—H12A	117.8
F1—C2—C1	120.05 (19)	C13—C12—H12A	117.8
F1—C2—C3	117.01 (18)	C11—C12—H12B	117.8
C1—C2—C3	122.9 (2)	C13—C12—H12B	117.8
N1—C3—C4	121.6 (2)	H12A—C12—H12B	114.9
N1—C3—C2	120.8 (2)	C11—C13—C12	60.01 (16)
C4—C3—C2	117.47 (18)	C11—C13—H13A	117.8
O1—C4—C3	116.36 (17)	C12—C13—H13A	117.8
O1—C4—C5	122.29 (18)	C11—C13—H13B	117.8
C3—C4—C5	121.36 (18)	C12—C13—H13B	117.8
N2—C5—C4	122.81 (18)	H13A—C13—H13B	114.9
N2—C5—C6	118.12 (17)	O4—C14—O3	122.4 (2)
C4—C5—C6	119.06 (18)	O4—C14—C9	125.0 (2)
C1—C6—C5	119.07 (18)	O3—C14—C9	112.52 (18)
C1—C6—C8	118.85 (18)	O3—C15—C16	108.7 (2)
C5—C6—C8	121.99 (18)	O3—C15—H15A	109.9
O1—C7—H7A	109.5	C16—C15—H15A	109.9
O1—C7—H7B	109.5	O3—C15—H15B	109.9
H7A—C7—H7B	109.5	C16—C15—H15B	109.9
O1—C7—H7C	109.5	H15A—C15—H15B	108.3
H7A—C7—H7C	109.5	C15—C16—H16A	109.5
H7B—C7—H7C	109.5	C15—C16—H16B	109.5
O2—C8—C9	124.20 (18)	H16A—C16—H16B	109.5
O2—C8—C6	120.70 (19)	C15—C16—H16C	109.5
C9—C8—C6	115.09 (17)	H16A—C16—H16C	109.5
C10—C9—C8	119.22 (18)	H16B—C16—H16C	109.5
C10—C9—C14	119.2 (2)	H1W—O1W—H2W	107 (3)
C8—C9—C14	121.54 (18)		

### Hydrogen-bond geometry (Å, °)

**Table:**

*D*—H···*A*	*D*—H	H···*A*	*D*···*A*	*D*—H···*A*
N1—H1N1···O1W^i^	0.91 (3)	2.15 (3)	2.930 (3)	143 (2)
N1—H2N1···O4^ii^	0.85 (3)	2.33 (3)	3.061 (3)	144 (2)
O1W—H1W···O2^iii^	0.84 (3)	2.13 (3)	2.916 (3)	155 (3)
O1W—H2W···O2	0.91 (3)	1.96 (3)	2.864 (3)	171 (3)

Symmetry codes: (i) −*x*+1, −*y*+1, −*z*+2; (ii) *x*, *y*, *z*+1; (iii) −*x*+1, −*y*+1, −*z*+1.

## References

[bb1] Farrugia, L. J. (1997). *J. Appl. Cryst.* **30**, 565.

[bb2] Fujita, M. & Chiba, K. (1998). *Chem. Pharm. Bull.* **46**, 631–638. 10.1248/cpb.46.631 9579039

[bb3] Gabe, E. J., Le Page, Y., Charland, J.-P., Lee, F. L. & White, P. S. (1989). *J. Appl. Cryst.* **22**, 384–387.

[bb4] Gabe, E. J. & White, P. S. (1993). *DIFRAC.* American Crystallographic Association Pittsburgh Meeting Abstract PA 104.

[bb5] Sheldrick, G. M. (2008). *Acta Cryst.* A**64**, 112–122. 10.1107/S0108767307043930 18156677

